# Clinical and Genetic Characteristics of Non-Insulin-Requiring Glutamic Acid Decarboxylase (GAD) Autoantibody-Positive Diabetes: A Nationwide Survey in Japan

**DOI:** 10.1371/journal.pone.0155643

**Published:** 2016-05-13

**Authors:** Junichi Yasui, Eiji Kawasaki, Shoichiro Tanaka, Takuya Awata, Hiroshi Ikegami, Akihisa Imagawa, Yasuko Uchigata, Haruhiko Osawa, Hiroshi Kajio, Yumiko Kawabata, Akira Shimada, Kazuma Takahashi, Kazuki Yasuda, Hisafumi Yasuda, Toshiaki Hanafusa, Tetsuro Kobayashi, Japan Diabetes Society Committee on Type 1 Diabetes Mellitus Research

**Affiliations:** 1 Department of Endocrinology and Metabolism, Nagasaki University Hospital, Nagasaki, Japan; 2 Department of Diabetes and Endocrinology, Shin-Koga Hospital, Kurume, Japan; 3 Third Department of Internal Medicine, University of Yamanashi, Yamanashi, Japan; 4 Department of Diabetes, Endocrinology and Metabolism, International University of Health and Welfare Hospital, Tochigi, Japan; 5 Department of Endocrinology, Metabolism and Diabetes, Kinki University Faculty of Medicine, Osaka, Japan; 6 Department of Metabolic Medicine, Graduate School of Medicine, Osaka University, Suita, Japan; 7 Diabetes Center, Tokyo Women's Medical University School of Medicine, Tokyo, Japan; 8 Department of Laboratory Medicine, Ehime University School of Medicine, Ehime, Japan; 9 Department of Diabetes, Endocrinology, and Metabolism, Center Hospital, National Center for Global Health and Medicine, Tokyo, Japan; 10 Department of Internal Medicine, Saiseikai Central Hospital, Tokyo, Japan; 11 Department of Diabetes and Metabolism, Iwate Medical University, Iwate, Japan; 12 Department of Metabolic Disorder, Diabetes Research Center, Research Institute, National Center for Global Health and Medicine, Tokyo, Japan; 13 Division of Health Sciences, Department of Community Health Sciences, Kobe University Graduate School of Health Sciences, Kobe, Japan; 14 Department of Internal Medicine (I), Osaka Medical College, Takatsuki, Japan; 15 Okinaka Memorial Institute for Medical Research, Tokyo, Japan; Baylor College of Medicine, UNITED STATES

## Abstract

**Aims:**

Glutamic acid decarboxylase autoantibodies (GADAb) differentiate slowly progressive insulin-dependent (type 1) diabetes mellitus (SPIDDM) from phenotypic type 2 diabetes, but many GADAb-positive patients with diabetes do not progress to insulin-requiring diabetes. To characterize GADAb-positive patients with adult-onset diabetes who do not require insulin therapy for >5 years (NIR-SPIDDM), we conducted a nationwide cross-sectional survey in Japan.

**Methods:**

We collected 82 GADAb-positive patients who did not require insulin therapy for >5 years (NIR-SPIDDM) and compared them with 63 patients with insulin-requiring SPIDDM (IR-SPIDDM). Clinical and biochemical characteristics, HLA-DRB1-DQB1 haplotypes, and predictive markers for progression to insulin therapy were investigated.

**Results:**

Compared with the IR-SPIDDM group, the NIR-SPIDDM patients showed later diabetes onset, higher body mass index, longer duration before diagnosis, and less frequent hyperglycemic symptoms at onset. In addition, C-peptide, LDL-cholesterol, and TG were significantly higher in the NIR-SPIDDM compared to IR-SPIDDM patients. The NIR-SPIDDM group had lower frequency of susceptible HLA-DRB1*04:05-DQB1*04:01 and a higher frequency of resistant HLA-DRB1*15:01-DQB1*06:02 haplotype compared to IR-SPIDDM. A multivariable analysis showed that age at diabetes onset (OR = 0.82), duration before diagnosis of GADAb-positive diabetes (OR = 0.82), higher GADAb level (≥10.0 U/ml) (OR = 20.41), and fasting C-peptide at diagnosis (OR = 0.07) were independent predictive markers for progression to insulin-requiring diabetes. An ROC curve analysis showed that the optimal cut-off points for discriminating two groups was the GADAb level of 13.6 U/ml, age of diabetes onset of 47 years, duration before diagnosis of 5 years, and fasting C-peptide of 0.65 ng/ml.

**Conclusions:**

Clinical, biochemical and genetic characteristics of patients with NIR-SPIDDM are different from those of IR-SPIDDM patients. Age of diabetes onset, duration before GADAb-positivity, GADAb level, and fasting C-peptide at diagnosis must be carefully considered in planning prevention trials for SPIDDM.

## Introduction

Type 1 diabetes (T1D) is caused by the autoimmune destruction of pancreatic islet β cells. Autoantibodies to glutamic acid decarboxylase (GADAb) are some of the major anti-islet autoantibodies, and a high prevalence of GADAb has been demonstrated in T1D patients [1–3]. GADAb is also detected in adult-onset patients initially diagnosed as having type 2 diabetes (T2D) who do not require insulin treatment but may become insulin-dependent within a few years after diagnosis. This subset of T1D is variably referred to as slowly progressive insulin-dependent (type 1) diabetes mellitus (SPIDDM) [4], latent autoimmune diabetes in adults (LADA) [5–7], or adult-onset autoimmune diabetes [8]. In the previous reports the prevalence of GADAb in healthy population has been reported to be 0.5–0.8% [9, 10], which is lower than that in non-insulin treated type 2 diabetes (T2D) (2–6%) and that in T2D who develop insulin dependency (11–15%) [11–15], supporting that GADAb is associated with the development of diabetes and insulin dependency.

There have been several studies seeking predictive markers of the progression to insulin-requiring diabetes among patients with SPIDDM. High levels of GADAb [8, 11, 16–17], antithyroid peroxidase antibody (TPOAb) [18], the presence of autoantibodies against the middle epitope of GAD65 and multiple anti-islet autoantibodies [19] have been reported to be predictive markers for insulin-requiring diabetes. However, there are a certain number of patients with GADAb-positive diabetes who do not progress to insulin-requiring diabetes for many years, and their clinical and genetic characteristics remain unclear.

In the present study therefore, we conducted a nationwide survey of diabetic patients with GADAb who had had non-insulin-requiring diabetes more than 5 years after the diagnosis, under the auspices of the Japan Diabetes Society.

## Subjects and Methods

### Patients

We asked all of the members of the Japan Diabetes Society through direct mail and the readership of the *Journal of the Japan Diabetes Society* whether they knew of candidates who have not required insulin treatment more than 5 years after the diagnosis of diabetes and whose GADAb test results were positive at some time point during the patient’s clinical course, termed as non-insulin-requiring SPIDDM (NIR-SPIDDM). The current diagnostic criteria for SPIDDM have been described elsewhere [20]. All subjects were of Japanese stock. We received positive responses from 25 hospitals. To those who responded positively, we sent questionnaires asking for a description of their candidates’ clinical characteristics and laboratory findings at the diagnosis of GADAb-positive diabetes, including the co-occurrence of autoimmune thyroid disease (AITD). We received data on the cases of 82 individuals (32 males, 50 females), including 2 cases who were already reported in the literature [21, 22]. These patients were reported from regions throughout Japan, from Hokkaido Island to Kyushu Island, and although the majority of cases were clustered in highly populated areas they were not restricted to any specific region or prefecture in Japan.

We also studied the cases of 63 individuals (18 males, 45 females) with insulin-requiring SPIDDM (IR-SPIDDM) who were consecutively recruited at Nagasaki University Hospital. The diagnosis of IR-SPIDDM was based on the following criteria: (1) the presence of GADAb at some time point during the patient’s clinical course and (2) the absence of ketosis or ketoacidosis at the onset (or diagnosis) of diabetes without the need for insulin treatment to correct hyperglycemia immediately after diagnosis, (3) no requirement for insulin treatment for at least 6 months after the diagnosis, and (4) the need for continuous insulin treatment for the management of hyperglycemia after the initiation of insulin. The initiation of insulin treatment was not influenced by awareness of autoantibody positivity. Acute or fulminant form of adult-onset type 1 diabetes were excluded.

Clinical AITD was defined as Graves’ disease (the presence of autoantibody to thyrotropin receptor) treated with antithyroid agents or Hashimoto thyroiditis (the presence of TPOAb and/or thyroglobulin antibodies) treated with hormone replacement therapy. The study was conducted according to the principles expressed in the Declaration of Helsinki. Participants did not provide their verbal or written informed consent to join the study, but were allowed to refuse participation. This procedure conforms to the Japanese Ethical Guidelines for Epidemiological Research; informed consent is not strictly required for observational studies using existing data. The study was approved by the ethics committee of the Japan Diabetes Society. Participant’s clinical records and laboratory data was anonymized and de-identified prior to analysis.

### Autoantibodies

Sera at some time point during each patient’s clinical course of NIR-SPIDDM and IR-SPIDDM were gathered from each hospital to the clinical laboratory centers and GADAb, insulinoma-associated antigen-2 autoantibodies (IA-2Ab), and insulin autoantibodies (IAA) were determined by a radioimmunoassay (RIA). The method for GADAb and IA-2Ab determination used in this study was RIA using ^125^I-labeled recombinant human GAD65 and IA-2, respectively, as a tracer reagent (RSR Ltd, Cardiff, UK) [23, 24]. IAA was determined by a fluid-phase RIA kit using competition with cold insulin and precipitation with polyethylene glycol with some modifications (Yamasa Corp, Chiba, Japan) [23]. The cut off value was 1.5 U/ml for the GADAb, 0.4 U/ml for the IA-2Ab, and 125nU/ml for IAA, respectively. The sensitivity/specificity values for GADAb and IA-2Ab were 82.6/93.6% and 66.0/98.9%, respectively, in the Diabetes Autoantibody Standardization Program 2009. In the 6th IAA Proficiency Workshop, IAA assay had a sensitivity, specificity, validity and consistency of 100%.

### HLA typing

HLA-DRB1 and HLA-DQB1 were genotyped by the polymerase chain reaction (PCR) sequence-specific primer and PCR sequence-specific oligonucleotide methods [25]. The most probable DRB1-DQB1 haplotypes were deduced from known linkage disequilibria. Allele frequencies were obtained by direct counting. We were able to determine the HLA haplotypes in 60 of the 82 (73%) cases with NIR-SPIDDM and 62 of the 63 (98%) patients with IR-SPIDDM. As a control for the analysis of HLA-DRB1 and HLA-DQB1 alleles, we also studied 304 unrelated healthy individuals (189 males, 115 females; median age 45.0 years; range 20.0–74.0 years) [26].

### Statistical analysis

The results are given as mean ± standard deviation (SD) or median (interquartile range) unless otherwise indicated. The statistical analyses were performed with a chi-square test and the Mann-Whitney *U* test. Laboratory data were obtained at the diagnosis of GADAb-positive diabetes. The significance of differences in the distribution of HLA-DRB1 and HLA-DQB1 alleles between the NIR-SPIDDM, IR-SPIDDM and healthy control groups was determined by a chi-square test. A multivariate logistic regression analysis was performed to analyze the predictive markers for insulin treatment. All variables were entered simultaneously into the model. Odds ratios (OR) and the 95% confidence interval (95%CI) were also calculated. A receiver operating characteristic (ROC) curve analysis was performed to calculate optimal cut-off point [27]. StatView (ver. 5.0; SAS Institute, Cary, NC) were used for these tests. P-values <0.05 were considered significant.

## Results

### Clinical characteristics and laboratory findings of NIR-SPIDDM patients

The clinical characteristics of the 82 patients with NIR-SPIDDM are summarized in Table 1. Their average age at diabetes onset was 54.0 ± 10.5 (mean ± SD) years, and the duration of diabetes was 14.0 ± 6.7 years. Among the 82 patients, insulin treatment was started in only six (7.3%) patients at more than 10 years after the diabetes onset. Their mean insulin-free period was 16.3 ± 4.5 years.

**Table 1:** Clinical characteristics of NIR-SPIDDM vs. IR-SPIDDM patients.

	NIR-SPIDDM	IR-SPIDDM	P-value
Cases	82	63	–
Females, n (%)	50 (61.0)	45 (71.4)	n.s.
Age at examination (yrs)	68.0±10.6	57.8±17.2	<0.001
Age at diagnosis of diabetes (yrs)	54.0±10.5	43.1±13.8	<0.0001
Duration of diabetes (yrs)	14.0±6.7	14.7±10.4	n.s.
Insulin-free period (yrs)	13.6±6.4	4.3±5.7	<0.0001
Duration before diagnosis of GADAb-positive diabetes (yrs)	9.4±7.4	6.7±8.9	<0.001
BMI at diagnosis as GADAb positive diabetes (kg/m^2^)	24.5±4.3	21.9±3.9	<0.0001
Maximum BMI (kg/m^2^)	27.8±3.7	25.6±4.3	<0.0001
Hyperglycemic symptoms at diabetes onset (%)	15 (18.3)	28 (44.4)	<0.001
Family history of diabetes (%)	49 (59.8)	39 (61.9)	n.s.
Co-occurrence of clinical AITD (%)	12 (14.6)	17 (27.0)	n.s.
Chronic thyroiditis	6 (7.3)	3 (4.8)	n.s.
Graves’ disease	6 (7.3)	14 (22.2)	<0.01
IA-2Ab (positive/negative)	3/9	11/21	n.s.
IAA (positive/negative)	3/8	1/10	n.s.

Data are n (%), or mean±SD. Hyperglycemic symptoms at diagnosis include thirst, polyuria, body weight loss. Clinical AITD is defined as indicated in *Subjects and Methods*. AITD, autoimmune thyroid disease; IAA, insulin autoantibodies; IA-2Ab, autoantibodies to insulinoma-associated antigen-2; n.s., not significant.

The patients’ median level of GADAb was 3.7 U/ml (range 1.5–14,000) and 63 of the 82 (76.8%) patients had GADAb levels lower than 10 U/ml (180 WHO U/ml) which has been reported as a cut-off value for the prediction of insulin requirement diabetes [7, 19, 28] (S1 Fig). Therefore, we divided these 82 cases into two groups according to their GADAb levels: <10 U/ml (n = 63) or ≥10 U/ml (n = 19) (S1 and S2 Tables). These groups’ median levels of GADAb were 2.8 U/ml (range 1.5–8.4) and 111.1 U/ml (10.3–14,000), respectively. The body mass index (BMI) and the triglyceride (TG) level at the diagnosis of GADAb-positive diabetes was significantly lower in the ≥10 U/ml patients compared to the corresponding values in the <10 U/ml patients (BMI 22.2 ± 4.6 vs. 25.2 ± 3.9 kg/m^2^, P<0.001, TG 99.7 vs. 134.5 ± 74.1 mg/dl, P<0.05). In addition, the maximum BMI tended to be lower in the ≥10 U/ml patients than in the <10 U/ml patients (25.7 ± 4.2 vs. 28.5 ± 3.3 kg/m^2^, P = 0.07).

### Comparison of NIR-SPIDDM and IR-SPIDDM patients

The predictive markers for the progression to insulin-requiring diabetes have been unclear. We therefore compared the clinical and laboratory findings between patients with NIR-SPIDDM and IR-SPIDDM. Although both the NIR-SPIDDM patients (males: females = 1: 1.56) and the IR-SPIDDM (males: females = 1: 2.50) patients were female-predominant, the difference between the female-to-male ratio in the NIR-SPIDDM and IR-SPIDDM groups was not significant.

In addition, the duration of diabetes was also similar between the NIR-SPIDDM and IR-SPIDDM groups. As shown in Table 1, the age at diabetes onset (54.0 ± 10.5 vs. 43.1 ± 13.8 years, P<0.0001), BMI at diagnosis as GADAb positive diabetes (24.5 ± 4.3 vs. 21.9 ± 3.9 kg/m^2^, P<0.0001), and maximum BMI (27.8 ± 3.7 vs. 25.6 ± 4.3 kg/m^2^, P<0.0001) were significantly higher in the NIR-SPIDDM patients compared to those in the IR-SPIDDM patients. Furthermore, the insulin-free period (13.6 ± 6.4 vs. 4.3 ± 5.7 years, P<0.0001) and the duration before the diagnosis of GADAb-positive diabetes (9.4 ± 7.4 vs. 6.7 ± 8.9 years, P<0.001) were significantly longer in the NIR-SPIDDM patients than in the IR-SPIDDM patients. The frequency of patients with hyperglycemic symptoms at diabetes onset was significantly lower in the NIR-SPIDDM patients (18.3 vs. 44.4%, P<0.001).

Data on IAA and/or IA-2Ab positivity were available in 18 patients with NIR-SPIDDM and 33 patients with IR-SPIDDM, respectively. Among these patients 4 NIR-SPIDDM and 15 IR-SPIDDM patients, respectively, were double autoantibody positive for IAA or IA-2Ab with GADAb. Two patients, one in IR-SPIDDM group and another in NIR-SPIDDM, were triple autoantibody positive.

### Comparison of laboratory findings and treatment at the diagnosis of GADAb-positive diabetes between the NIR-SPIDDM and IR-SPIDDM patients

As shown in Table 2, the HbA1c (8.0 ± 1.7% vs. 10.5 ± 2.7%, P<0.0001), plasma glucose (162.9 ± 64.0 vs. 238.2 ± 93.6 mg/dl, P<0.01), and GADAb levels (3.7 [1.5–14000] vs. 90.1 [2.1–144000] U/ml, P<0.0001) were significantly lower in the NIR-SPIDDM patients compared to the IR-SPIDDM patients. However, the fasting serum C-peptide (FCPR) (1.9 ± 0.7 vs. 0.8 ± 0.9 ng/ml, P<0.0001), postprandial serum C-peptide (PCPR) (3.4 ± 1.9 vs. 1.6 ± 1.7 ng/ml, P<0.0001), LDL-cholesterol (110.8 ± 29.0 vs. 109.2 ± 36.8 mg/dl, P<0.05), TG (126.2 ± 72.1 vs. 91.1 ± 41.0 mg/dl, P<0.01), and uric acid (4.9 ± 1.3 vs. 4.2 ± 1.3 mg/dl, P<0.001) were higher in the NIR-SPIDDM patients than those in the IR-SPIDDM patients. Although the prevalence of patients treated with oral hypoglycemic agents was significantly higher in NIR-SPIDDM, the proportion of patients taking each agent was similar between the two groups. Forty-nine percent of IR-SPIDDM patients had been started insulin treatment at the diagnosis of GADAb-positive diabetes. None of the patients were negative for urinary ketons.

**Table 2:** Laboratory findings and treatment of NIR-SPIDDM vs. IR-SPIDDM patients at the diagnosis of GADAb-positive diabetes.

	NIR-SPIDDM	IR-SPIDDM	P-value
HbA1c (%)	8.0±1.7	10.5±2.7	<0.0001
Plasma glucose (mg/dl)	162.9±64.0	238.2±93.6	<0.01
FCPR (ng/ml)	1.9±0.7	0.8±0.9	<0.0001
PCPR (ng/ml)	3.4±1.9	1.6±1.7	<0.0001
GADAb level (U/ml)	3.7 (1.5–14000)	90.1 (2.1–144000)	<0.0001
Total cholesterol (mg/dl)	193.0±35.1	190.1±44.8	<0.05
HDL-cholesterol (mg/dl)	55.8±14.4	61.0±18.8	n.s.
LDL-cholesterol (mg/dl)	110.8±29.0	109.2±36.8	<0.05
Triglyceride (mg/dl)	126.2±72.1	91.1±41.0	<0.01
Uric Acid (mg/dl)	4.9±1.3	4.2±1.3	<0.001
Treatment			
Diet/Exercise only	18/82 (22.0)	13/63 (20.6)	n.s.
Oral hypoglycemic agents	64/82 (78.0)	19/63 (30.2)	<0.0001
Sulfonylurea	46/64 (71.9)	13/19 (68.4)	n.s.
Glinide	5/64 (7.8)	0/19 (0.0)	n.s.
DPP-4 inhibitor	10/64 (15.6)	3/19 (15.8)	n.s.
Biguanide	26/64 (40.6)	4/19 (21.1)	n.s.
Thiazolidine	12/64 (18.8)	2/19 (10.5)	n.s.
α-glucosidase inhibitor	19/64 (29.7)	7/19 (36.8)	n.s.
Insulin	0/82 (0.0)	31/63 (49.2)	<0.0001

Data are n (%), mean±SD or median (range). FCPR, Fasting serum C-peptide; PCPR, postprandial serum C-peptide; n.s., not significant.

### HLA-DRB1 and HLA-DQB1 haplotype and genotype frequencies

We next examined whether or not the NIR-SPIDDM and IR-SPIDDM groups have different genetic backgrounds. Table 3 provides the frequencies of the HLA-DRB1 and HLA-DQB1 haplotype in the patients with NIR-SPIDDM, those with IR-SPIDDM, and the healthy controls. Among the susceptible haplotypes in these Japanese patients with T1D, the HLA DRB1*04:05-DQB1*04:01 haplotype was more frequent in both the NIR-SPIDDM (19.2%) and IR-SPIDDM (30.6%) patients compared to the controls (12.2%). In addition, this haplotype was significantly less frequent in the NIR-SPIDDM patients compared to the IR-SPIDDM patients (P<0.05). The major protective haplotype in Japanese patients with T1D, the HLA DRB1*15:01-DQB1*06:02 haplotype, was significantly less frequent in the IR-SPIDDM group than in both the NIR-SPIDDM patients and the healthy controls (P<0.01).

**Table 3:** Frequency of the HLA DRB1-DQB1 haplotype in NIR-SPIDDM, IR-SPIDDM, and healthy controls.

DRB1-DQB1	NIR-SPIDDM	IR-SPIDDM	Healthy controls	NIR-SPIDDM vs.IR-SPIDDM		IR-SPIDDM vs. controls		NIR-SPIDDM vs. controls	
	(n = 120)	(n = 124)	(n = 608)	P-value	OR (95% CI)	P-value	OR (95% CI)	P-value	OR (95% CI)
*01:01–*05:01	4 (3.3)	5 (4.0)	35 (5.8)	n.s.	−	n.s.	−	n.s.	−
*04:03–*03:02	7 (5.8)	3 (2.4)	21 (3.5)	n.s.	−	n.s.	−	n.s.	−
*04:05–*04:01	23 (19.2)	38 (30.6)	74 (12.2)	<0.05	1.86 (1.03–3.37)	<0.0001	3.19 (2.03–5.01)	<0.05	1.71(1.02–2.87)
*04:06–*03:02	5 (4.2)	2 (1.6)	20 (3.3)	n.s.	−	n.s.	−	n.s.	−
*08:02–*03:02	4 (3.3)	6 (4.8)	13 (2.2)	n.s.	−	n.s.	−	n.s.	−
*08:03–*06:01	10 (8.3)	7 (5.6)	65 (10.7)	n.s.	−	n.s.	−	n.s.	−
*09:01–*03:03	19 (15.8)	28 (22.6)	100 (16.4)	n.s.	−	n.s.	−	n.s.	−
*13:02–*06:04	2 (1.7)	9 (7.3)	21 (3.5)	n.s.	—	n.s.	−	n.s.	−
*15:01–*06:02	10 (8.3)	1 (0.8)	48 (7.9)	<0.01	0.09 (0.01–0.71)	<0.01	0.09 (0.01–0.69)	n.s.	−
*15:02–*06:01	9 (7.5)	6 (4.8)	57 (9.4)	n.s.	−	n.s.	−	n.s.	−
Others	27 (22.5)	19 (15.3)	154 (25.3)						

Data are n (%). Data whose total frequencies were less than 3.0% in each group were excluded. n.s., not significant; OR, Odds ratios

CI, confidence interval.

Furthermore, HLA DRB1*04:05-DQB1*04:01/ DRB1*09:01-DQB1*03:03 genotype was significantly more frequent in the IR-SPIDDM patients compared to both the NIR-SPIDDM patients and the controls (P<0.05, S3 Table). Any HLA DRB1-DQB1 haplotype or genotype was not associated with GADAb levels.

### Predictive markers for the progression to insulin-requiring diabetes

We performed a multivariate logistic regression analysis to determine the predictive markers for the progression to insulin-requiring diabetes (Table 4). This analysis demonstrated that the age at diabetes onset (OR 0.82, 95%CI 0.73–0.94; P<0.005), the duration before the diagnosis of GADAb-positive diabetes (OR 0.82, 95%CI 0.69–0.98; P<0.05), higher levels of GADAb (≥10.0 U/ml) (OR 20.41, 95%CI 1.75–238.57; P<0.05), FCPR at the diagnosis of GADAb-positive diabetes (OR 0.07, 95%CI 0.01–0.65; P<0.05), but neither susceptible nor protective HLA haplotypes, were found to be the predictors of progression to the insulin-requiring diabetes.

**Table 4:** Multivariate logistic regression analysis for predictive markers of progression to insulin-requiring diabetes.

Variable	OR	95% CI	P-value
Females	7.16	0.48–107.31	n.s.
Co-occurrence of clinical AITD	0.16	0.00–7.33	n.s.
Hyperglycemic symptoms at diabetes onset	24.14	0.61–957.47	n.s.
Age at diabetes onset	0.82	0.73–0.94	<0.005
Duration before diagnosis of GADAb-positive diabetes	0.82	0.69–0.98	<0.05
BMI at diagnosis of GADAb-positive diabetes	0.99	0.74–1.32	n.s.
GADAb ≥10.0 U/ml	20.41	1.75–238.57	<0.05
HbA1c at diagnosis of GADAb-positive diabetes	1.55	0.66–3.65	n.s.
FCPR at diagnosis of GADAb-positive diabetes	0.07	0.01–0.65	<0.05
Susceptible HLA haplotype	0.18	0.01–3.07	n.s.
Resistant HLA haplotype	0.18	0.01–2.13	n.s.

All variables were entered simultaneously into the model. Clinical AITD is defined as indicated in *Subjects and Methods*. Hyperglycemic symptoms are thirst, polyuria, and body weight loss. AITD, autoimmune thyroid disease; FCPR, Fasting serum C-peptide; n.s., not significant; OR, Odds ratio; CI, confidence interval. Susceptible HLA haplotype = DRB1*04:05-DQB1*04:01, DRB1*08:02-DQB1*03:02, DRB1*09:01-DQB1*03:03; Resistant HLA haplotype = DRB1*15:01-DQB1*06:02, DRB1*15:02-DQB1*06:01, DRB1*08:03-DQB1*06:01.

### The optimal cut-off value of the predictive markers for progression to the insulin-requiring stage

We calculated the optimal cut-off value of GADAb levels for the progression to insulin-requiring diabetes by conducting a ROC curve analysis of the NIR-SPIDDM and IR-SPIDDM groups (Table 5). The optimal cut-off level of GADAb was 13.6 U/ml (77.8% sensitivity, 81.7% specificity) in the overall patients. Because it has been reported that the coexistence of AITD affects the GADAb levels, we divided the NIR-SPIDDM and IR-SPIDDM patients into two groups, patients with clinical AITD [AITD(+)] and without AITD [AITD(−)]. The calculated optimal cut-off value was higher in the AITD(+) group (28.0 U/ml, 88.2% sensitivity, 91.7% specificity vs. 13.6 U/ml, sensitivity 71.7%, 82.9% specificity), confirming the association between the presence of AITD and GADAb levels.

**Table 5:** Optimal cut-off values of GADAb level, age at diabetes onset, duration before diagnosis of GADAb-positive diabetes, and FCPR for a progression to insulin-requiring diabetes by ROC curve analysis.

		NIR-SPIDDM/ IR-SPIDDM	Cut-off value	Sensitivity	Specificity	AUC
GADAb level	All subjects	82/63	13.6 U/ml	77.8%	81.7%	0.837
	AITD (+)	12/17	28.0 U/ml	88.2%	91.7%	0.902
	AITD (−)	70/46	13.6 U/ml	71.7%	82.9%	0.815
Age at diabetes onset	All subjects	82/63	47 yrs	60.3%	78.0%	0.728
Duration before diagnosis of GADAb-positive diabetes	All subjects	82/63	5 yrs	65.1%	67.1%	0.670
FCPR at diagnosis of GADAb positive diabetes	All subjects	82/63	0.65 ng/ml	61.4%	97.6%	0.852

Data are n, or %. FCPR, Fasting serum C-peptide; AUC, area under the curve; AITD, autoimmune thyroid disease.

Because the patients’ age at diabetes onset, duration before diagnosis of GADAb-positive diabetes, and FCPR at diagnosis of GADAb-positive diabetes were shown to be predictive markers in addition to GADAb levels, we also determined the optimal cut-off value of these parameters for discriminating NIR-SPIDDM and IR-SPIDDM by ROC curve analysis (Table 5). The optimal cut-off values of these factors were 47 years (60.3% sensitivity, 78.0% specificity), 5 years (65.1% sensitivity, 67.1% specificity), and 0.65 ng/ml (61.4% sensitivity, 97.6% specificity), respectively.

## Discussion

Here we conducted a nationwide retrospective survey to analyze the clinical, biochemical and genetic characteristics of Japanese patients with SPIDDM who did not require insulin treatment for more than 5 years (NIR-SPIDDM), and we compared these characteristics with those of patients with IR-SPIDDM.

It has been reported that a GADAb level ≥10 U/ml (≥180 WHO U/ml) is one of the predictive markers for the progression to insulin-requiring diabetes in SPIDDM [7, 19, 28]. However, we observed no remarkable differences in clinical and laboratory findings except for BMI between our two patient groups classified according to their GADAb levels, <10 U/ml or ≥10 U/ml, among NIR-SPIDDM patients (S1 and S2 Tables). These results suggest that NIR-SPIDDM patients are a unique and relatively homogeneous subgroup among diabetic patients with GADAb. Although Zampetti S et al. [29] found that insulin sensitizers maintained the insulin-free period longer than sulfonylureas in GADAb-positive patients with diabetes, it is unlikely that specific oral hypoglycemic agents could influence the risk of progression toward insulin treatment in our patients.

Compared to the IR-SPIDDM patients, the NIR-SPIDDM patients had higher ages at their diabetes onset, and at their diagnoses of GADAb-positive diabetes, they had higher FCPR levels, longer durations before the diagnosis of GADAb-positive diabetes, and lower GADAb levels (Tables 1 and 2), and these factors were emerged as the predictive markers for the progression to insulin-requiring diabetes by multivariable analysis (Table 4). Although number of patients who were also measured IAA and/or IA-2Ab in NIR-SPIDDM patients (22.0%) was smaller than that in IR-SPIDDM patients (52.4%), the prevalence of multiple anti-islet autoantibody-positive patients was not significantly different between two groups (27.8% vs. 30.6%). These results lead to the speculation that underlying pathogenesis of NIR-SPIDDM are distinct from those of IR-SPIDDM and multiple islet autoantibody positivity might not be a risk factor for the progression to insulin dependency in NIR-SPIDDM.

The optimal cut-off values of GADAb levels and age at diabetes onset were 13.6 U/ml and 47 years, respectively (Table 5). These values were similar to the previous reports in Japanese or Caucasian patients [28, 30]. Although Murao and coworkers reported that positive TPOAb and IA-2Ab may contribute to the progression to β-cell failure in Japanese patients with LADA [18], in our present study multiple islet autoantibody positivity and the coexistence of clinical AITD was not found to be a predictor of insulin-requiring diabetes. However, the optimal cut-off value of GADAb was higher in the SPIDDM patients with AITD compared to the patients without AITD, which is consistent with our previous study [31]. These results suggest that the elevation of GADAb observed in SPIDDM patients with AITD might not be associated with islet β-cell destruction.

It was reported that the genetic background of Japanese T1D patients differs from that of Caucasian T1D patients [32]. Three HLA DRB1-DQB1 haplotypes, i.e., DRB1*04:05-DQB1*04:01, DRB1*08:02-DQB1*03:02, and DRB1*09:01-DQB1*03:03, confer susceptibility to Japanese T1D. Moreover, the DRB1*15:02-DQB1*06:01 haplotype, which is rare in Caucasoid populations, is a major protective haplotype, in addition to the DRB1*15:01-DQB1*06:02 haplotype, which is also protective in Caucasians [25].

In the present study, the DRB1*04:05-DQB1*04:01 haplotype, but not DRB1*08:02-DQB1*03:02 or DRB1*09:01-DQB1*03:03, was significantly more frequent in both the NIR-SPIDDM and IR-SPIDDM patients compared to the healthy controls. Of note, this result indicates that the NIR-SPIDDM who might have type 2 diabetes due to false positive assay still showed significant HLA risk. The DRB1*04:05-DQB1*04:01 haplotype, especially in combination with DRB1*09:01-DQB1*03:03, may thus confer the susceptibility to SPIDDM. In regard to protective haplotypes, DRB1*15:01-DQB1*06:02 was significantly less frequent in the present IR-SPIDDM patients than in both the NIR-SPIDDM patients and controls. These results suggest that a protective haplotype may help interfere with the progression to insulin-requiring diabetes in SPIDDM patients.

Although several studies have investigated potential predictive markers for future insulin requirement in adult-onset autoimmune diabetes [19, 28, 29, 33], to our knowledge there has been no study investigating such markers in patients with GADAb-positive diabetes who do not progress to insulin-requiring diabetes for a long time. Our present findings demonstrated that younger age at the diagnosis of diabetes, shorter duration before the diagnosis of GADAb-positive diabetes, a GADAb level ≥13.6 U/ml, and a lower FCPR level at the diagnosis of GADAb-positive diabetes were useful predictive markers to differentiate progressors from long-term non-progressors among GADAb-positive diabetic patients. These results, in part, are in accordance with previous data [12, 30], and should be carefully considered in planning future prevention trials for SPIDDM.

Our study has several limitations. First, the study was designed as a retrospective study, and thus the period from the diagnosis of diabetes to the examination of the GADAb level and the timing of initiation of insulin treatment were left to the treating physicians. Second, the number of patients was relatively small despite the multi-center nature of the study. Third, other islet autoantibodies, such as IAA, IA-2Ab, and zinc transporter-8 autoantibodies (ZnT8Ab), were either available in small number of patients or not at all for ZnT8A. A prospective study using a large number of subjects is required to establish the predictive markers for the progression to insulin-requiring diabetes in SPIDDM patients.

In conclusion, our present results demonstrated the clinical, biochemical and genetic characteristics of NIR-SPIDDM, including predictive markers for the progression to insulin-requiring diabetes. These novel findings may be a key to understanding the pathogenesis of both NIR-SPIDDM and IR-SPIDDM.

## Supporting Information

S1 Fig Distribution of GADAb levels in the NIR-SPIDDM patients (n = 82). Black bars indicate the number of patients in each GADAb level group.
(TIF)

S1 File The list of the doctors who referred the patients to our committee. (PDF)

S1 Table Clinical characteristics of NIR-SPIDDM patients with low versus high GADAb level. Data are n (%), or mean±SD. Hyperglycemic symptoms at diagnosis include thirst, polyuria, and body weight loss. Clinical AITD is defined as indicated in the *Subjects and Methods* section. AITD, autoimmune thyroid disease; n.s., not significant.
(PDF)

S2 Table Laboratory findings and treatment of NIR-SPIDDM patients with low vs. high GADAb levels at the diagnosis of GADAb-positive diabetes. Data are n (%), mean±SD or median (range). FCPR, Fasting serum C-peptide; PCPR, postprandial serum C-peptide; n.s., not significant.
(PDF)

S3 Table Frequency of the HLA DRB1-DQB1 haplotype combination in NIR-SPIDDM, IR-SPIDDM, and healthy controls. Data are n (%). X = any DRB1-DQB1 haplotype but *04:05-*04:01, *08:02-*03:02, *09:01-*03:03, *15:01-*06:02, or *15:02-*06:01; Z = any DRB1-DQB1 haplotype; n.s., not significant; OR, Odds ratios; CI, confidence interval.
(PDF)
