# Supplementary figures and images for: Clinical and Genetic Characteristics of Non-Insulin-Requiring Glutamic Acid Decarboxylase (GAD) Autoantibody-Positive Diabetes: A Nationwide Survey in Japan
Source: PLoS One. 2016 May 13;11(5):e0155643. doi: 10.1371/journal.pone.0155643 (PMC4866691; doi:10.1371/journal.pone.0155643)

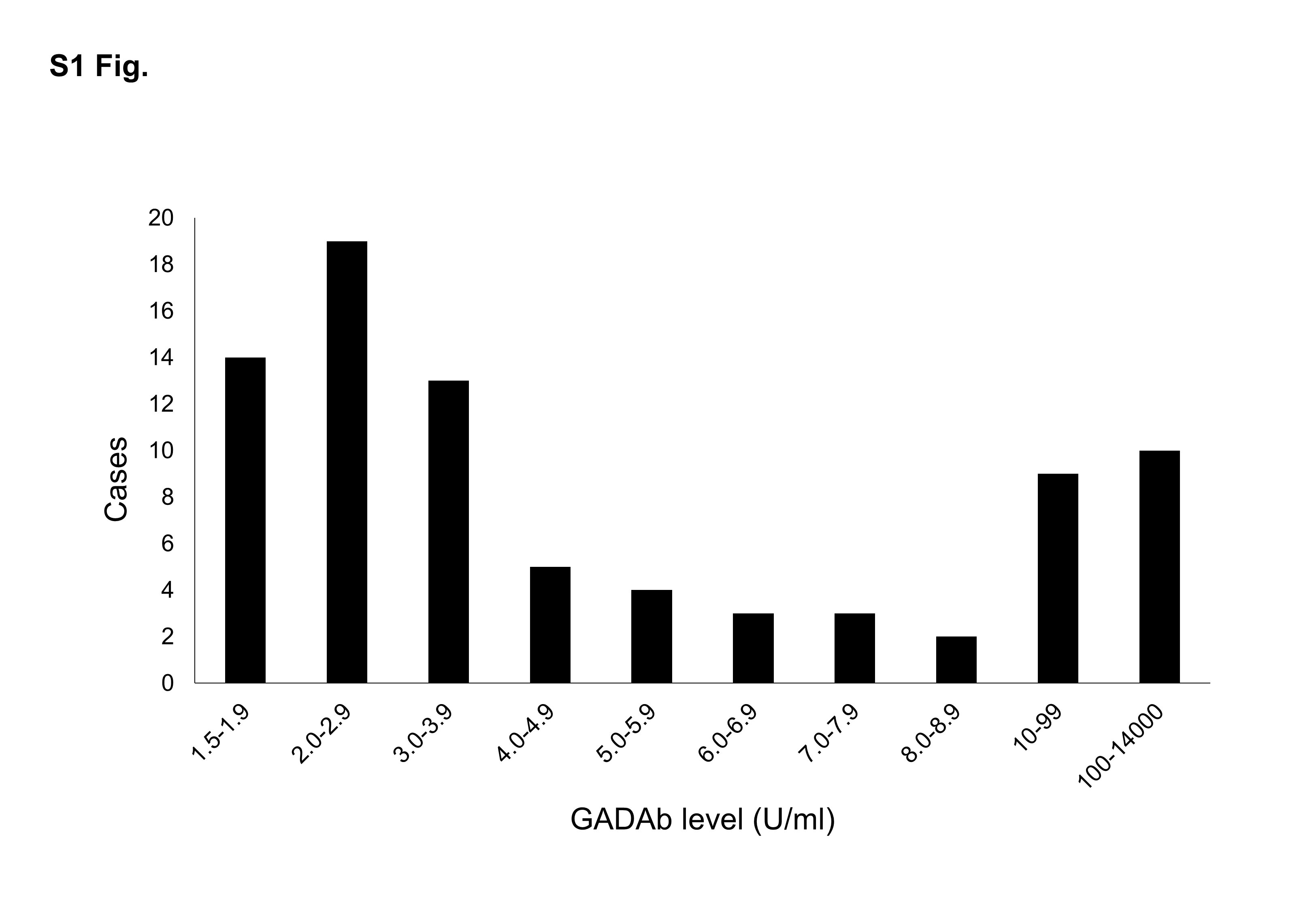

Supplement: S1 Fig — Black bars indicate the number of patients in each GADAb level group. (TIF) [file pone.0155643.s001.tif]
