# Supplementary material for: Clinical and Genetic Characteristics of Non-Insulin-Requiring Glutamic Acid Decarboxylase (GAD) Autoantibody-Positive Diabetes: A Nationwide Survey in Japan
Source: PLoS One. 2016 May 13;11(5):e0155643. doi: 10.1371/journal.pone.0155643 (PMC4866691; doi:10.1371/journal.pone.0155643)
Supplement: S1 File — (PDF) [file pone.0155643.s002.pdf]

The list of the doctors who referred the patients to our committee.

We extend our sincere appreciation to the following doctors who referred the patients to our committee: K. Aida (The Third Department of Internal Medicine, Faculty of Medicine, The University of Yamanashi); T. Fukuda (Department of Diabetes, Sakakibara Heart Institute of Okayama); Y. Hayashi (Department of Endocrinology and Metabolism, Kariya Toyota General Hospital); M. Hiramatsu and T. Inoue (Department of Diabetes, Metabolism and Endocrinology, Fukuoka City Medical Association Hospital); K. Hirao (H.E.C Science Clinic); Y. Hiromine (Department of Endocrinology, Metabolism and Diabetes, Kinki University Faculty of Medicine); C. Horiguchi and J. Eguchi (Diabetes Center, Okayama University Hospital); C. Inada (Inada Clinic); A. Iwahashi (Department of Diabetes and Endocrinology, The Japanese Red Cross Wakayama Medical Center); M. Izaki (Izaki Clinic); K. Kaneko and K. Kamoi (Department of Diabetes, Endocrinology and Metabolism, Nagaoka Red Cross Hospital); Y. Kawabata (Department of Endocrinology, Metabolism and Diabetes, Kinki University Faculty of Medicine); K. Kawai (Kawai Clinic); H. Kitaoka and M. Kure (Department of Internal Medicine, Keiseikai Hospital); Y. Kishitani (Department of Gastroenterology and Endocrinology, Nara Hospital Kinki University Faculty of Medicine); Y. Kubota (Department of Diabetes, Nakadori Hospital); Y. Kurihara (Kurihara Clinic); K. Matsumoto (Diabetes Center, Sasebo Chuo Hospital); S. Murao (Department of Diabetes and Endocrinology, Takamatsu Hospital); K. Nagashima (Department of Diabetes and Clinical Nutrition, Kyoto University

Hospital); T. Sawa (Department of Internal Medicine, Kawasakisaiwai Hospital); A. Takeda (Department of Diabetes and Metabolism, Shinko Hospital); K. Takeshima (Department of Endocrinology and Rheumatology, Kurashiki Central Hospital); Y. Ueda (The Japanese Red Cross Nagasaki Genbaku Hospital); T. Yamada (Department of Diabetes and Metabolism, Tohoku University Hospital); S. Yamamoto (Yuri Clinic); H. Yanai (Department of Internal Medicine, Kohnodai Hospital, National Center for Global Health and Medicine).
