# Supplementary material for: Clinical and Genetic Characteristics of Non-Insulin-Requiring Glutamic Acid Decarboxylase (GAD) Autoantibody-Positive Diabetes: A Nationwide Survey in Japan
Source: PLoS One. 2016 May 13;11(5):e0155643. doi: 10.1371/journal.pone.0155643 (PMC4866691; doi:10.1371/journal.pone.0155643)
Supplement: S1 Table — Data are n (%), or mean±SD. Hyperglycemic symptoms at diagnosis include thirst, polyuria, and body weight loss. Clinical AITD is defined as indicated in the Subjects and Methods section. AITD, autoimmune thyroid disease; n.s., not significant. (PDF) [file pone.0155643.s003.pdf]

S1 Table. Clinical characteristics of NIR-SPIDDM patients with low versus high GADAb level

	<10 U/ml	≥10 U/ml	P-value
Cases	63	19	-
Males, n (%)	27 (42.9)	5 (26.3)	n.s.
Age at diabetes onset (yrs)	53.4±9.6	56.2±12.6	n.s.
Duration of diabetes (yrs)	14.1±6.8	13.4±6.2	n.s.
Insulin-free period (yrs)	13.7±6.5	13.3±6.2	n.s.
Duration before diagnosis of GADAb-positive diabetes (yrs)	9.6±7.3	8.7±7.4	n.s.
BMI at diagnosis as GADAb-positive diabetes (kg/m^2)	25.2±3.9	22.2±4.6	<0.001
Maximum BMI (kg/m^2)	28.5±3.3	25.7±4.2	0.070
Hyperglycemic symptoms at diabetes onset (%)	12 (19.0)	3 (15.8)	n.s.
Family history of diabetes (%)	37 (58.7)	12 (63.2)	n.s.
Co-occurrence of clinical AITD (%)	8 (12.7)	4 (21.1)	n.s.

Data are n (%), or mean±SD.

Hyperglycemic symptoms at diagnosis include thirst, polyuria, and body weight loss.

Clinical AITD is defined as indicated in the *Research Design and Methods* section.

AITD, autoimmune thyroid disease; n.s., not significant.
