# Supplementary material for: Clinical and Genetic Characteristics of Non-Insulin-Requiring Glutamic Acid Decarboxylase (GAD) Autoantibody-Positive Diabetes: A Nationwide Survey in Japan
Source: PLoS One. 2016 May 13;11(5):e0155643. doi: 10.1371/journal.pone.0155643 (PMC4866691; doi:10.1371/journal.pone.0155643)
Supplement: S2 Table — Data are n (%), mean±SD or median (range). FCPR, Fasting serum C-peptide; PCPR, postprandial serum C-peptide; n.s., not significant. (PDF) [file pone.0155643.s004.pdf]

S2 Table. Laboratory findings and treatment of NIR-SPIDDM patients with low vs. high GADAb levels at the diagnosis of GADAb-positive diabetes

	<10 U/ml	≥10 U/ml	P-value
HbA1c (%)	8.1±1.8	7.9±1.5	n.s.
Plasma glucose (mg/dl)	168.6±67.4	144.9±47.8	n.s.
FCPR (ng/ml)	1.9±0.7	1.8±0.7	n.s.
PCPR (ng/ml)	3.4±1.9	3.5±2.0	n.s.
GADAb level (U/ml)	2.8 (1.5-8.4)	111.1 (10.3-14000)	<0.0001
Total cholesterol (mg/dl)	196.6±33.3	181.3±38.4	n.s.
HDL-cholesterol (mg/dl)	56.4±14.1	53.8±15.2	n.s.
LDL-cholesterol (mg/dl)	112.4±27.7	105.7±32.3	n.s.
Triglyceride (mg/dl)	134.5±74.1	99.7±58.0	<0.05
Uric Acid (mg/dl)	4.9±1.3	4.6±1.3	n.s.
Treatment			
Diet/Exercise only	15/63 (23.8)	3/19 (15.8)	n.s.
Oral hypoglycemic agents	48/63 (76.2)	16/19 (84.2)	n.s.
Sulfonylurea	36/48 (75.0)	10/16 (62.5)	n.s.
Glinide	2/48 (4.2)	3/16 (18.8)	n.s.
DPP-4 inhibitor	8/48 (16.7)	2/16 (12.5)	n.s.
Biguanide	23/48 (47.9)	3/16 (18.8)	<0.05
Thiazolidine	10/48 (20.8)	2/16 (12.5)	n.s.
α-glucosidase inhibitor	13/48 (27.1)	6/16 (37.5)	n.s.

Data are n (%), mean±SD or median (range). FCPR, Fasting serum C-peptide; PCPR, postprandial serum C-peptide; n.s., not significant
