# Supplementary material for: Clinical and Genetic Characteristics of Non-Insulin-Requiring Glutamic Acid Decarboxylase (GAD) Autoantibody-Positive Diabetes: A Nationwide Survey in Japan
Source: PLoS One. 2016 May 13;11(5):e0155643. doi: 10.1371/journal.pone.0155643 (PMC4866691; doi:10.1371/journal.pone.0155643)
Supplement: S3 Table — Data are n (%). X = any DRB1-DQB1 haplotype but *04:05-*04:01, *08:02-*03:02, *09:01-*03:03, *15:01-*06:02, or *15:02-*06:01; Z = any DRB1-DQB1 haplotype; n.s., not significant; OR, Odds ratios; CI, confidence interval. (PDF) [file pone.0155643.s005.pdf]

**S3 Table. Frequency of the HLA DRB1-DQB1 haplotype combination in NIR-SPIDDM, IR-SPIDDM, and healthy controls**

| DRB1-DQB1 | NIR-SPIDDM | IR-SPIDDM | Healthy controls | NIR-SPIDDM vs.IR-SPIDDM | | IR-SPIDDM vs. controls | | NIR-SPIDDM vs. controls | |
|---|---|---|---|---|---|---|---|---|---|
| | (n=60) | (n=62) | (n=304) | P-value | OR (95% CI) | P-value | OR (95% CI) | P-value | OR (95% CI) |
| *04:05-04:01/*04:05-04:01 | 2 (3.3) | 5 (8.1) | 3 (1.0) | n.s. | – | <0.001 | 8.80 (2.05-37.9) | n.s. | – |
| *04:05-04:01/*08:02-*03:02 | 2 (3.3) | 3 (4.8) | 4 (1.3) | n.s. | – | n.s. | – | n.s. | – |
| *04:05-04:01/*09:01-03:03 | 3 (5.0) | 10 (16.1) | 17 (5.6) | <0.05 | 3.65 (0.95-14.0) | <0.005 | 3.25 (1.41-7.48) | n.s. | – |
| *04:05-04:01/X | 13 (21.7) | 12 (19.4) | 38 (12.5) | n.s. | – | n.s. | – | n.s. | – |
| *08:02-*03:02/*08:02-*03:02 | 0 | 0 | 1 (0.3) | – | – | – | – | – | – |
| *08:02-*03:02/*09:01-0303 | 2 (3.3) | 1 (1.6) | 1 (0.3) | n.s. | – | n.s. | – | <0.05 | 10.4 (0.93-117.1) |
| *08:02-*03:02/X | 1 (1.7) | 2 (3.2) | 14 (4.6) | n.s. | – | n.s. | – | n.s. | – |
| *09:01-0303/*09:01-0303 | 3 (5.0) | 1 (1.6) | 10 (3.3) | n.s. | – | n.s. | – | n.s. | – |
| *09:01-0303/X | 6 (10.0) | 13 (21.0) | 43 (14.1) | n.s. | – | n.s. | – | n.s. | – |
| *15:01–*06:02/Z or *15:02–*06:01/Z | 19 (31.7) | 7 (11.3) | 100 (32.9) | <0.01 | 0.27 (0.11-0.71) | <0.001 | 0.26 (0.11-0.59) | n.s. | – |
| X/X | 9 (15.0) | 8 (12.9) | 73 (24.0) | n.s. | – | n.s. | – | n.s. | – |

Data are n (%). X = any DRB1-DQB1 haplotype but *04:05-*04:01, *08:02-*03:02, *09:01-*03:03, *15:01-*06:02, or *15:02-*06:01; Z = any DRB1-DQB1 haplotype; n.s., not significant; OR, Odds ratios; CI, confidence interval.
